# Estradiol Triggers Cerebellar MLI-PC LTP via ERβ/Protein Kinase C Signaling Cascades in Mice In Vivo

**DOI:** 10.3390/ijms26209973

**Published:** 2025-10-14

**Authors:** Zhao-Yi Zhang, Li Chen, Ming-Ze Sun, Chao-Yue Chen, Chun-Yan Wang, Yuki Todo, Zheng Tang, Yan-Cong Lv, Qin-Yong Zou, Chun-Ping Chu, Yin-Hua Xu, De-Lai Qiu

**Affiliations:** 1 Department of Physiology and Pathophysiology, College of Medicine, Yanbian University, Yanji 133002, Chinacpchu@ybu.edu.cn (C.-P.C.); 2 Department of Neural Circuit, Institute of Brain Science, Jilin Medical University, Jilin 132013, Chinachenchaoyue5480@163.com (C.-Y.C.); qinyongzou@gmail.com (Q.-Y.Z.); 3 Department of Physiology, College of Basic Medicine, Jilin Medical University, Jilin 132013, China; 4 Faculty of Electrical, Information and Communication Engineering, Kanazawa University, Kanazawa 920-1192, Ishikawa, Japan; 5 School of Computer Engineering and Science, Shanghai University, Shanghai 200444, China; 6 Department of Neurology, College of Medicine, Yanbian University, Yanji 133002, China

**Keywords:** estrogen receptors, cerebellar molecular layer interneuron–Purkinje cell synaptic transmission, in vivo cell-attached recording, sensory stimulation, protein kinase C (PKC)

## Abstract

17β-estradiol (E2) enhances the cerebellar molecular layer interneurons (MLIs)—Purkinje cells (PCs) synaptic transmission via activation of the Erβ in vivo in mice. Whether E2 regulates cerebellar MLI-PC synaptic plasticity is unknown. To investigate the mechanism of E2, we evaluated the modulation of facial stimulation-evoked MLI-PC long-term plasticity in mice. Cell-attached recordings from PCs of Crus II were performed using an Axopatch-700B patch-clamp amplifier. The MLI-PC synaptic transmission was evoked by facial stimulation. Immunohistochemistry was used to detect the expression of ERβ. Under control conditions, 1 Hz facial stimuli induced long-term depression (LTD) at MLI-PC synapses, characterized by a sustained reduction in P1 amplitude and a simple spike (SS) pause. The facial stimulus-induced MLI-PC LTD was completely prevented by E2, but this effect was reversed by a selective ERα/ERβ antagonist, ICI182780. Blockade of cannabinoid receptor 1 (CB1R) eliminated the MLI-PC LTD under control conditions, but revealed an E2-triggered long-term potentiation (LTP). The E2-triggered MLI-PC LTP persisted in the presence of an ERα antagonist but was absent in the presence of an ERβ antagonist PHTPP. The E2-triggered MLI-PC LTP remained unaffected by protein kinase A inhibition but was abolished by inhibition of protein kinase C (PKC) and intracellular Ca^2+^ depletion. Moreover, ERβ immunoreactivity was abundantly distributed around dendrites and somas of PCs in the Crus II region of the mouse cerebellar cortex. The present results suggest that E2 activates ERβ, thereby triggering facial stimulation-induced MLI-PC LTP via the PKC signaling cascade, which occludes CB1R-dependent MLI-PC LTD in the cerebellar cortex of mice in vivo.

## 1. Background

In the cerebellar cortical circuit, long-term synaptic plasticity has been demonstrated at the parallel fiber–Purkinje cell (PF-PC), PF molecular layer interneurons (MLs), mossy fiber–granule cells (MF-GCs), and MLI-PC synapses [1,2,3,4]. Significantly, the long-term plasticity of the MLI-PC synapses can be induced by activating the heterosynaptic cannabinoid receptor 1 (CB1R) and N-Methyl-D-Aspartate receptors (NMDARs) within the isolated mouse cerebellum [2,3]. Under in vivo conditions, facial stimulation at a frequency of 1 Hz has the ability to induce MLI-PC long-term depression (LTD), which is also contingent upon the activities of NMDARs and CB1R cerebellar cortex in mice [5]. MLI-PC LTD, like other forms of long-term synaptic plasticity in the cerebellar cortex, is considered a potential mechanism underlying motor learning [1,2,3,4,5].

The neuromodulatory effects of 17β-estradiol (E2) in the brain have been widely investigated, and these effects involve sexual behavior, neural synaptic plasticity, and cognition [6]. In the cerebellar cortex, estrogen receptor α (ERα) and estrogen receptor β (ERβ) are widely expressed, with prominent expression in the granular cell layer and molecular layer. Recently, we demonstrated immunoreactivity for ERβ in the molecular layer (ML) and Purkinje cell (PC) layer of the mouse cerebellar cortex, with particular enrichment around PC somas [7]. Estrogens potentiate PC responsiveness to glutamate [8] and enhance glutamatergic synaptic transmission through activation of ERs in cerebellar slices of adult rats [8,9,10]. Under in vivo conditions, cerebellar surface application of E2 produces an enhancement in the cerebellar MLI-PC synaptic transmission and an improvement of the initial motor learning ability of mice through Erβ [7]. Importantly, the activity-dependent regulation of the dendritic spine cytoskeleton, LTP induction, and memory formation have been shown to depend on the activation of Erβ [11,12]. In addition, E2 augments NMDAR-mediated excitatory post-synaptic potentials and facilitates hippocampal LTP in both awake rats [13] and cultured hippocampal slices [14]. The persistent enhancement of synaptic strength, a defining feature of long-term potentiation (LTP), is widely regarded as a key indicator of neuronal synaptic plasticity. Activation of ERα triggers the emergence of LTP in gamma-aminobutyric acidergic (GABAergic) synaptic transmission within the oval bed nucleus of the stria-terminalis in rats [15], and induces an NMDAR-dependent LTP at temporoammonic-hippocampal CA1 synapses of rats in vitro [16]. In female mice, subcutaneous administration of estradiol can enhance cerebellar cortical PF-PC LTP and promote synapse formation by activating ERβ, thereby improving motor skills [17]. In addition, blocking the synthesis of estradiol can rapidly impair the increase and decrease in the gain in the process of vestibulo-ocular reflex adaptation without changing the performance of the basic oculomotor function [18].

Since both ERα and ERβ are widely expressed in the cerebellar cortex, and E2 enhances cerebellar MLI-PC synaptic transmission via activation of ERβ, we hypothesized that E2 modulates MLI-PC long-term synaptic plasticity in the cerebellar cortex through activation of ER subtypes and their downstream signaling cascades. Therefore, we investigated the mechanism by which E2 modulates the long-term synaptic plasticity of MLI—PC synapses induced by facial sensory stimulation in the mouse cerebellar cortex in vivo.

## 2. Results

### 2.1. E2 Occluded the 1 Hz Facial Stimulation-Evoked MLI-PC LTD via the Activation of ERs

**Figure 1:**
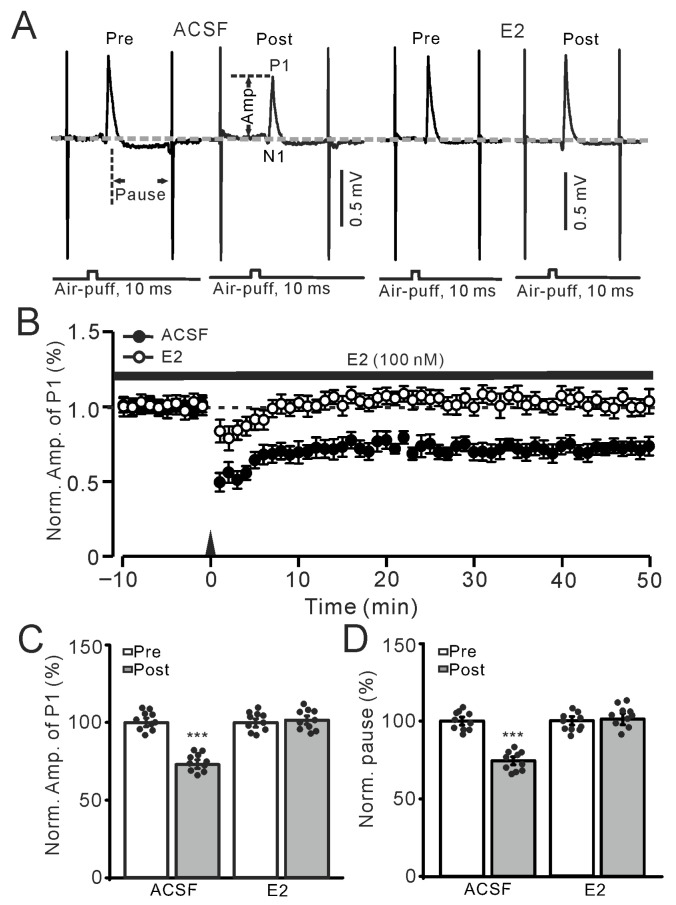
Estradiol (E2) impaired the facial stimulation-induced molecular layer interneuron–Purkinje cell long-term depression (MLI-PC LTD) in the mouse cerebellar cortex. (**A**) Representative membrane potentials show the air-puff stimulation evoked-MLI-PC response (P1) before (Pre) and after (Post) the delivery of 1 Hz (240 pulses) stimulation during treatment with artificial cerebrospinal fluid (ACSF, left) and E2 (100 nM; right). The measurements of P1 amplitude (P1 Amp) and SS pause duration are shown in the figure. (**B**) A summary of data showing the normalized amplitude of P1 over time in the ACSF group (solid circles) and the E2 group (hollow circles) before and after the delivery of 1 Hz stimulation (black arrow). Note that facial stimulation induced MLI-PC LTD in ACSF, which was absent in the presence of E2. (**C**) The bar graph with individual data shows the normalized P1 amplitude before (Pre) and after (Post) 1 Hz stimulation during treatment with ACSF (Pre: 100.0 ± 1.5%, Post: 74.3 ± 1.2%, F (1,18) = 240.3, *p* < 0.001; *n* = 10 recordings/10 mice), and E2 (Pre: 100.0 ± 1.7%, Post: 101.5 ± 2.1%, F (1,18) = 0.48, *p* = 0.51; *n* = 10 recordings/10 mice). (**D**) Bar graph with individual data shows the normalized simple spike (SS) pause time before (Pre) and after (Post) 1 Hz stimulation during treatment with ACSF (Pre: 100.0 ± 1.4%, Post: 74.3 ± 1.2%, F (1,18) = 190.7, *p* < 0.001; *n* = 10 recordings/10 mice), and E2 (Pre: 100.0 ± 1.3%, Post: 101.2 ± 2.7%, F (1,18) = 0.45, *p* = 0.63; *n* = 10 recordings/10 mice). *** *p* < 0.001 versus Pre. Note that facial stimulation induced a decrease in P1 amplitude and SS pause duration in ACSF, but no such decrease was observed in the presence of E2.

Consistent with previous studies [5,19], air-puff stimulation of the ipsilateral whisker pad at 1 Hz (240 pulses) could induce MLI-PC LTD (Figure 1A,B), which exhibited a significant decrease in the amplitude of P1 and the simple spike pause for more than 50 min. The amplitude of P1 during 40–50 min after 1 Hz stimulation in the control (ACSF) group was 75.8 ± 1.0% of that before the induction (*p* < 0.001; *n* = 10; Figure 1C). In addition, the SS pause during 40–50 min after 1 Hz stimulation in the control (ACSF) group was 74.3 ± 1.2% of that before the induction (*p* < 0.001; *n* = 10; Figure 1D). However, perfusion of E2 (100 nM) to the cerebellar surface completely prevented the 1 Hz air-puff stimulation-induced MLI-PC LTD (Figure 1A,B). In the presence of E2, the amplitude of P1 during 40–50 min after 1 Hz stimulation was 101.5 ± 2.1% of that before the induction (*p* = 0.51; *n* = 10; Figure 1C), and the SS pause during 40–50 min after 1 Hz stimulation was 101.2 ± 2.7% of that before the induction (*p* = 0.63; *n* = 10; Figure 1D). These results indicate that 1 Hz facial stimulation induces a cerebellar MLI-PC LTD, which is occluded by cerebellar surface application of E2 in vivo in mice.

**Figure 2:**
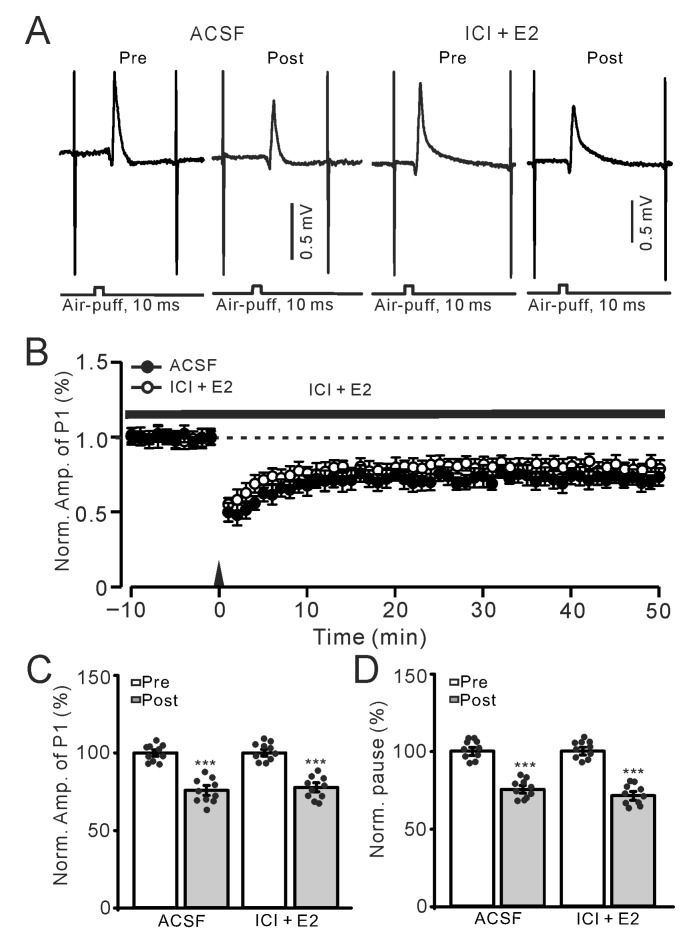
Application of a nonselective ER antagonist, ICI (100 nM), eliminated the effect of E2 on the facial stimulation-evoked MLI-PC LTD. (**A**) Representative membrane potentials show air-puff stimulation-evoked MLI-PC response (P1) before (Pre) and after (Post) the delivery of 1 Hz stimulation during treatment with ACSF (left) and ICI182780 (ICI, 100 nM) + E2 (100 nM; right). (**B**) A summary of data showing the normalized amplitude of P1 over time in the ACSF group (solid circles) and the ICI + E2 group (hollow circles) before and after the delivery of an air-puff stimuli train (black arrow). (**C**) The bar graph with individual data shows the normalized P1 amplitude before (Pre) and after (Post) 1 Hz stimulation during treatment with ACSF (Pre: 100.0 ± 1.0%, Post: 74.7 ± 1.5%, F (1,18) = 210.7, *p* < 0.001; *n* = 10 recordings/10 mice) and ICI + E2 (Pre: 100.0 ± 0.8%, Post: 76.2 ± 1.4%, F (1,18) = 152.4, *p* < 0.0001; *n* = 10 recordings/10 mice). (**D**) The bar graph with individual data shows the normalized SS pause time before (Pre) and after (Post) 1 Hz stimulation during treatment with ACSF (Pre: 100.0 ± 1.2%, Post: 75.3 ± 1.5%, F (1,18) = 0.36, *p* = 0.77; *n* = 10 recordings/10 mice) and ICI + E2 (Pre: 100.0 ± 1.4%, Post: 75.3 ± 1.5%, F (1,18) = 196.1, *p* < 0.001; *n* = 10 recordings/10 mice). *** *p* < 0.001 versus Pre. Note that E2 failed to prevent the facial stimulation-evoked MLI-PC LTD in the presence of an ER antagonist.

We next used the non-selective estrogen receptor antagonist ICI182780 (ICI) to investigate whether E2 occluded MLI-PC LTD by activating ERs. In the presence of ICI (100 nM), application of E2 (100 nM) did not occlude the 1 Hz facial stimulation-induced MLI-PC LTD (Figure 2A,B). In the presence of ICI and E2, the amplitude of P1 during 40–50 min after 1 Hz stimulation was 76.2 ± 1.4% of that before the induction (*p* < 0.001; *n* = 10; Figure 2C), which was similar to that in ACSF (*p* = 0.65; *n* = 10; Figure 2C). The SS pause during 40–50 min after 1 Hz stimulation was 75.3 ± 1.5% of that before the induction (*p* < 0.001; *n* = 10; Figure 2D), which was not significantly different than that in ACSF (*p* = 0.77; *n* = 10; Figure 2D). These results indicate E2 occludes the facial stimulation-induced MLI-PC LTD in the mouse cerebellar cortex by activating ERs.

### 2.2. Blockade of CBR1-Dependent LTD: E2 Triggered 1 Hz Facial Stimulation-Induced MLI-PC LTP in Mouse Cerebellar Cortex Through Activation of ERβ

**Figure 3:**
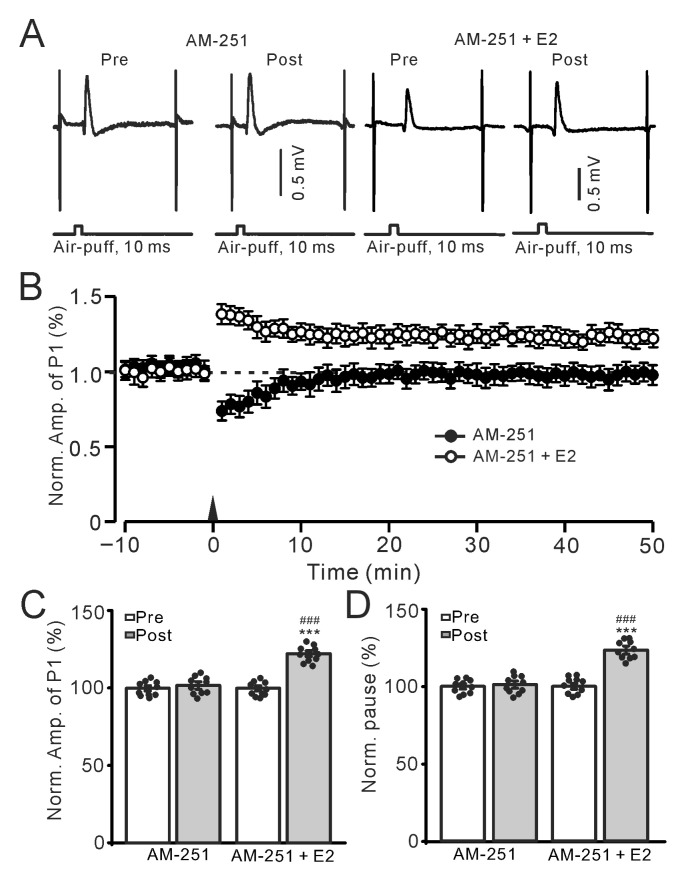
In the presence of AM-251, E2 triggered the facial stimulation-induced MLI-PC LTP. (**A**) Representative membrane potentials showing air-puff stimulation evoked MLI-PC response before (Pre) and after (Post) delivery of the 1 Hz (240 pulses) stimulation during treatment with AM-251 (5 μM control; left) and AM-251 (5 μM) + E2 (100 nM; right). (**B**) A summary of data showing the normalized amplitude of P1 over time in the AM-251 group (solid circles) and the AM-251 + E2 group (open circles) before and after the air-puff stimuli train (black arrow). (**C**) The bar graph with individual data shows the normalized P1 amplitude before (Pre) and after (Post) 1 Hz stimulation during treatment with AM-251 (Pre: 100.0 ± 1.2%, Post: 101.4 ± 1.4%, F (1,18) = 0.41, *p* = 0.53; *n* = 10 recordings/10 mice) and AM-251 + E2 (Pre: 100.0 ± 1.2%, Post: 122.4 ± 1.3%, F (1,18) = 295.7, *p* < 0.001; *n* = 10 recordings/10 mice). (**D**) The bar graph with individual data shows the normalized SS pause time before (Pre) and after (Post) 1 Hz stimulation during treatment with AM-251 (Pre: 100.0 ± 1.2%, Post: 101.1 ± 1.3%, F (1,18) = 0.36, *p* = 0.65; *n* = 10 recordings/10 mice) and AM-251 + E2 (Pre: 100.0 ± 1.3%, Post: 123.4 ± 1.6%, F (1,18) = 305.1, *p* < 0.001; *n* = 10 recordings/10 mice). *** *p* < 0.001 versus Pre; ^###^ *p* < 0.001 versus Post of AM-251. Note that E2 triggered the facial stimulation-induced MLI-PC LTP in the presence of a CB1R antagonist.

Since the induction of the MLI-PC LTD in the mouse cerebellar cortex requires the activation of cannabinoid receptor 1 (CB1R) [5], we further employed a CB1R antagonist, AM-251, to observe the effect of E2 on the 1 Hz sensory stimulation-induced MLI-PC synaptic plasticity. In the presence of AM-251 (5 μM), air-puff stimulation at 1 Hz (240 pulses) failed to induce the MLI-PC LTD in the control group, but the stimulation train induced MLI-PC long-term potentiation (LTP) in cerebellar cortex (Figure 3A,B). In the presence of AM-251 alone, the amplitude of P1 was 101.4 ± 1.4% of that before the induction (*p* = 0.53; *n* = 10; Figure 3C), and the SS firing pause was 101.1 ± 1.3% of that before the induction (*p* = 0.65; *n* = 10; Figure 3D). In the presence of the mixture of AM-251 and E2, the amplitude of P1 was 122.4 ± 1.3% of that before the induction (*p* < 0.001; *n* = 10; Figure 3C), which was significantly different than that in AM-251 alone (*p* < 0.05; *n* = 10; Figure 3D). In addition, the SS firing pause was 123.4 ± 1.6% of that before the induction (*p* < 0.001; *n* = 10; Figure 3D), which was also significantly higher than that in the AM-251 alone (*p* < 0.05; *n* = 10; Figure 3D). These results indicate that under the condition of blocking the CB1R-dependent MLI-PC LTD, E2 triggers 1 Hz facial stimulation-induced MLI-PC LTP in the mouse cerebellar cortex in vivo.

**Figure 4:**
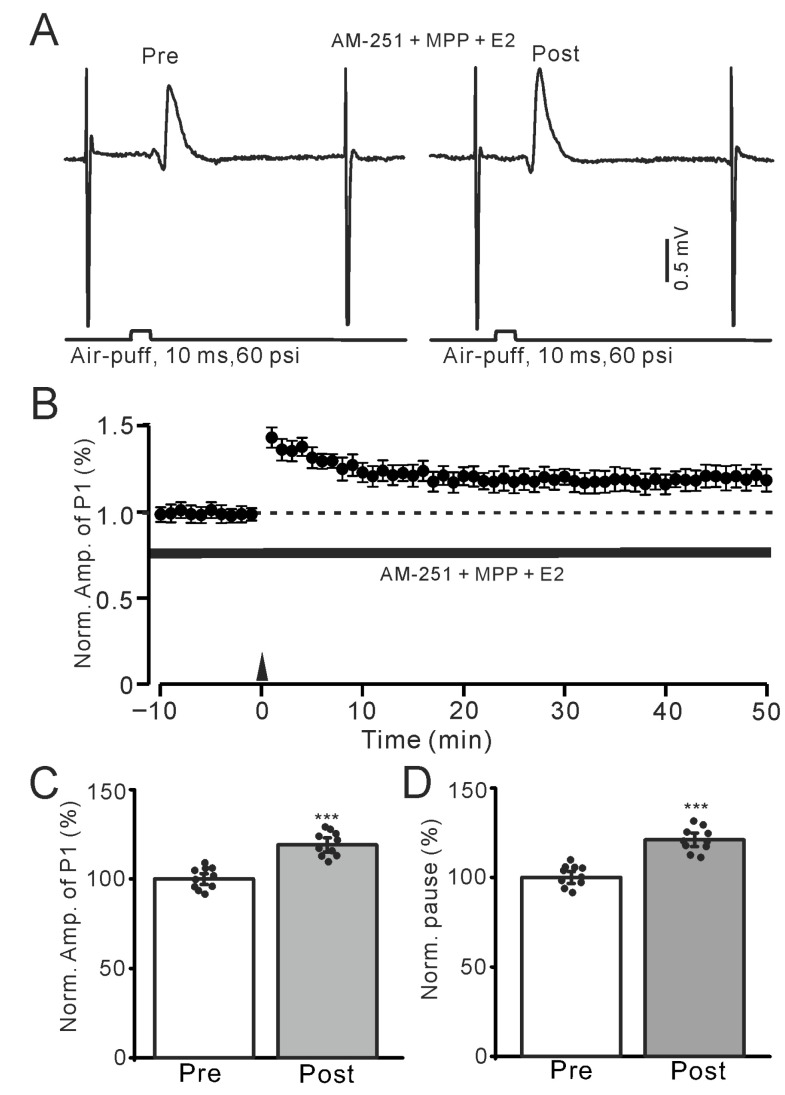
In the presence of an ERα antagonist and AM-251, E2 triggered the facial stimulation-induced MLI-PC LTP. (**A**) Representative membrane potentials showing air-puff stimulation evoked MLI-PC response before (Pre) and after (Post) delivery of 1 Hz (240 pulses) facial stimulation during treatment AM-251 (5 μM) + MPP (100 nM) + E2 (100 nM). (**B**) A summary of data showing the normalized amplitude of P1 in the presence of AM-251 + MPP + E2 before and after the air-puff stimuli train (black arrow). (**C**) The bar graph with individual data shows the normalized P1 amplitude before (Pre) and after (Post) 1 Hz stimulation in the presence of AM-251 + MPP + E2 (Pre: 100.0 ± 2.9%, Post: 119.1 ± 3.8%, F (1,18) = 236.4, *p* < 0.001; *n* = 10 recordings/10 mice). (**D**) The bar graph with individual data shows the normalized SS pause time before (Pre) and after (Post) 1 Hz stimulation in the presence of AM-251 + MPP + E2 (Pre: 100.0 ± 3.2%, Post: 121.1 ± 3.7%, F (1,18) = 275.6, *p* < 0.001; *n* = 10 recordings/10 mice). *** *p* < 0.001 versus Pre. Note that the E2-triggered MLI-PC LTP persisted in the presence of an ERα antagonist.

**Figure 5:**
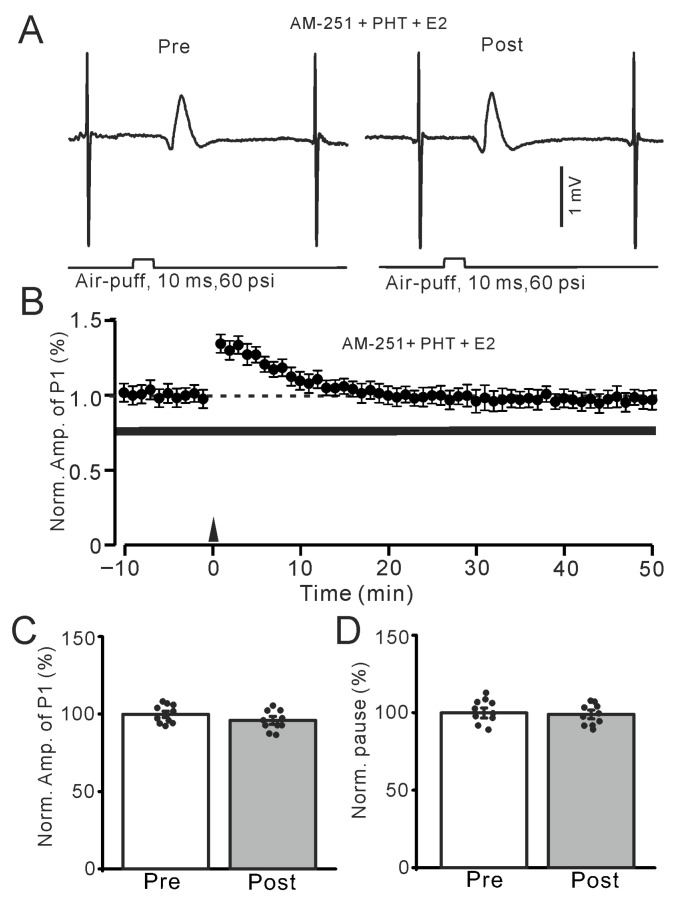
In the presence of an ERβ antagonist and AM-251, E2 failed to trigger the facial stimulation-induced MLI-PC LTP. (**A**) Representative membrane potentials showing air-puff stimulation evoked MLI-PC response before (Pre) and after (Post) delivery of the 1 Hz (240 pulses) stimulation during treatment with AM-251 (5 μM) + PHT (100 nM) + E2 (100 nM). (**B**) A summary of data showing the normalized amplitude of P1 in the presence of AM-251 + PHT + E2 before and after the air-puff stimuli train (black arrow). (**C**) Bar graph with individual data shows the normalized P1 amplitude before (Pre) and after (Post) 1 Hz stimulation during treatment with a mixture of AM-251 + PHT + E2 (Pre: 100.0 ± 2.0%, Post: 97.1 ± 2.6%, F (1,18) = 1.65, *p* = 0.36; *n* = 10 recordings/10 mice). (**D**) Bar graph with individual data shows the normalized SS pause time before (Pre) and after (Post) 1 Hz stimulation during treatment with a mixture of AM-251 + PHT + E2 (Pre: 100.0 ± 3.2%, Post: 99.1 ± 2.7%, F (1,18) = 1.26, *p* = 0.67; *n* = 10 recordings/10 mice). Note that the E2-triggered MLI-PC LTP was abolished by the blockade of ERβ.

We further employed the selective ERα antagonist MPP (100 nM) to investigate whether E2 triggered the facial stimulation-induced MLI-PC LTP by activating ERα. In the presence of AM-251 (5 μM) and MPP, E2 could still trigger the facial stimulation-induced MLI-PC (Figure 4A,B). The amplitude of P1 was 119.1 ± 3.8% of that before the induction (*p* < 0.001; *n* = 10; Figure 4C), and the SS pause was 121.1 ± 3.7% of that before the induction (*p* < 0.001; *n* = 10; Figure 4D). The results indicate that the application of the selective ERα antagonist MPP does not affect the E2-triggered facial stimulation-induced MLI-PC LTP in vivo in mice. Subsequently, we used the selective ERβ antagonist PHTPP (100 nM) to study whether E2 triggered the 1 Hz facial stimulation-induced MLI-PC LTP by activating ERβ. In the presence of AM-251 and PHTPP, E2 failed to trigger the facial stimulation-induced MLI-PC (Figure 5A,B). The amplitude of P1 was 97.1 ± 2.6% of that before the induction (*p* = 0.36; *n* = 10; Figure 5C), and the SS pause was 99.1 ± 2.7% of that before the induction (*p* = 0.67; *n* = 10; Figure 5D). The results indicate that the application of the selective ERβ antagonist PHTPP, but not the selective ERα antagonist MPP, completely prevented the E2-triggered facial stimulation-induced MLI-PC LTP in vivo in mice. These results suggest that E2 triggers the facial stimulation-induced MLI-PC LTP in the mouse cerebellar cortex by activating ERβ but not ERα.

**Figure 6:**
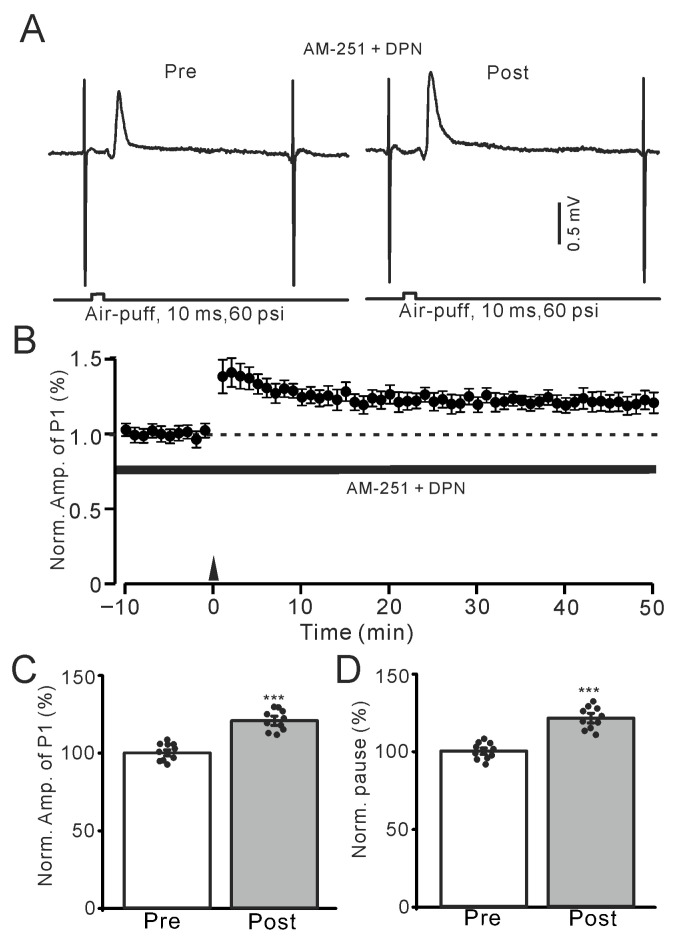
Application of an ERβ agonist, DPN, triggered the facial stimulation-induced MLI-PC LTP. (**A**) Representative membrane potentials showing air-puff stimulation evoked MLI-PC response before (Pre) and after (Post) delivery of the 1 Hz (240 pulses) stimulation during treatment AM-251 (5 μM) + DPN (100 nM). (**B**) A summary of data showing the normalized amplitude of P1 in the presence of AM-251 + DPN before and after the air-puff stimuli train (black arrow). (**C**) The bar graph with individual data shows the normalized P1 amplitude before (Pre) and after (Post) 1 Hz stimulation during treatment with AM-251 + DPN (Pre: 100.0 ± 1.9%, Post: 120.1 ± 2.9%, F (1,18) = 231.6, *p* < 0.001; *n* = 10 recordings/10 mice). (**D**) The bar graph with individual data shows the normalized SS pause time before (Pre) and after (Post) 1 Hz stimulation during treatment with AM-251 + PHT + E2 (Pre: 100.0 ± 2.1%, Post: 121.3 ± 3.1%, F (1,18) = 253.5, *p* < 0.001; *n* = 10 recordings/10 mice). *** *p* < 0.001 versus Pre. Note that pharmacological activation of ERβ triggered MLI-PC LTP.

The above results indicate that E2 triggers facial stimulation-induced MLI-PC LTP in the mouse cerebellar cortex by activating ERβ, suggesting that pharmacological activation of ERβ may also induce this form of MLI-PC LTP. To confirm that facial stimulation-evoked MLI-PC LTP can be triggered by ERβ activation, we further employed a selective ERβ agonist, DPN (100 nM), to investigate whether selective activation of ERβ could trigger 1 Hz facial stimulation-induced MLI-PC LTP in the mouse cerebellar cortex. In the presence of AM-251, application of DPN (100 nM) triggered the facial stimulation-induced MLI-PC LTP (Figure 6A,B). Specifically, the amplitude of P1 was 120.1 ± 2.9% of that before induction (*p* < 0.001; *n* = 10; Figure 6C), and the SS pause was 121.3 ± 3.1% of that before induction (*p* < 0.001; *n* = 10; Figure 6D). The results indicate that the pharmacological activation of ERβ triggers the facial stimulation-induced MLI-PC LTP in the mouse cerebellar cortex. The results suggested that E2 triggers the MLI-PC LTP in the mouse cerebellar cortex induced by activating ERβ, thereby occluding the expression of MLI-PC LTD.

### 2.3. E2 Triggered the 1 Hz Facial Stimulation-Induced MLI-PC LTP in Mouse Cerebellar Cortex Through PKC Signaling Cascade

**Figure 7:**
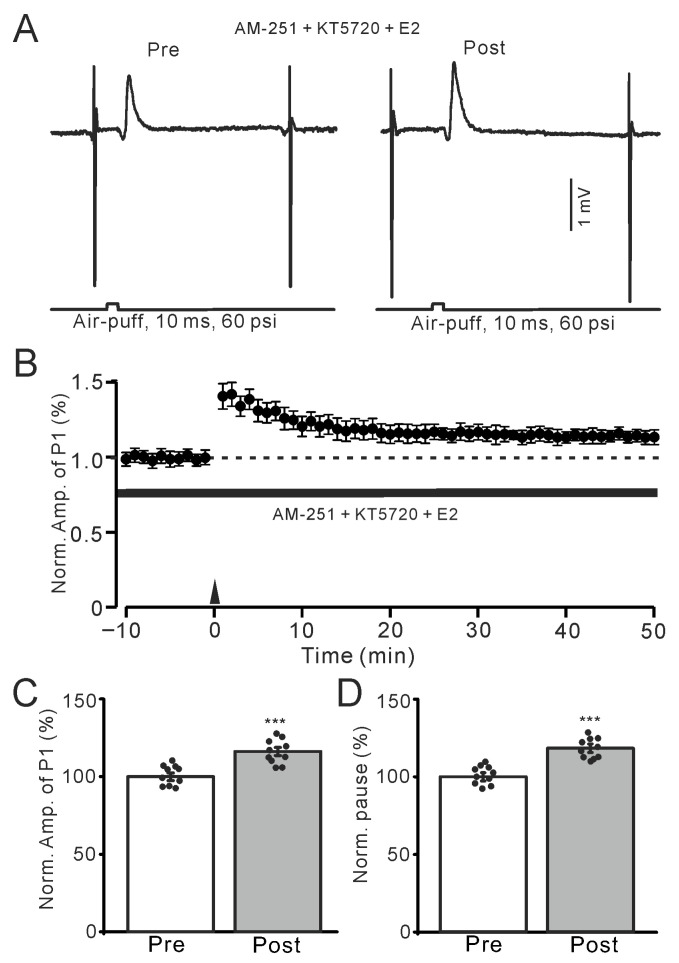
In the presence of KT-5720 and AM-251, E2 still triggered the facial stimulation-induced MLI-PC LTP. (**A**) Representative membrane potentials showing air-puff stimulation evoked MLI-PC response before (Pre) and after (Post) delivery of the 1 Hz (240 pulses) stimulation during treatment with a mixture of KT5720 (100 nM) + AM-251 (5 μM) + E2 (100 nM). (**B**) A summary of data showing the normalized amplitude of P1 in the presence of KT5720 + AM-251 + E2 before and after the air-puff stimuli train (black arrow). (**C**) The bar graph with individual data shows the normalized P1 amplitude before (Pre) and after (Post) 1 Hz stimulation during treatment with KT5720 + AM-251 + E2 (Pre: 100.0 ± 2.5%, Post: 116.3 ± 2.6%, F (1,18) = 97.8, *p* < 0.001; *n* = 10 recordings/10 mice). (**D**) The bar graph with individual data shows the normalized SS pause time before (Pre) and after (Post) 1 Hz stimulation during treatment with a mixture of KT5720 + AM-251 + E2 (Pre: 100.0 ± 2.6%, Post: 118.4 ± 2.7%, F (1,18) = 112.6, *p* < 0.001; *n* = 10 recordings/10 mice). *** *p* < 0.001 versus Pre. Note that inhibition of PKA failed to prevent the E2-triggered MLI-PC LTD in mouse cerebellar cortex in vivo.

Previous studies have demonstrated that E2 binding to ER can activate several signaling kinases, including protein kinase A (PKA) and protein kinase C (PKC) [20]. Therefore, we used a specific membrane-permeable PKA inhibitor, KT-5720 (100 nM), to investigate whether E2 triggers the MLI-PC LTP by activating the PKA signaling pathway. Inhibition of PKA with KT5720 did not prevent the E2-triggered MLI-PC LTP (Figure 7A,B). In the presence of AM-251 and KT5720, the amplitude of P1 was 116.3 ± 2.6% of that before the induction (*p* < 0.001; *n* = 10; Figure 7C), and the SS pause was 118.4 ± 2.7% of that before the induction (*p* < 0.001; *n* = 10; Figure 7D). The results indicate that even with the inhibition of PKA, E2 still triggers the facial stimulation-induced MLI-PC LTP, suggesting that the PKA signaling pathway is not mediated the E2-triggered MLI-PC in vivo in mice.

**Figure 8:**
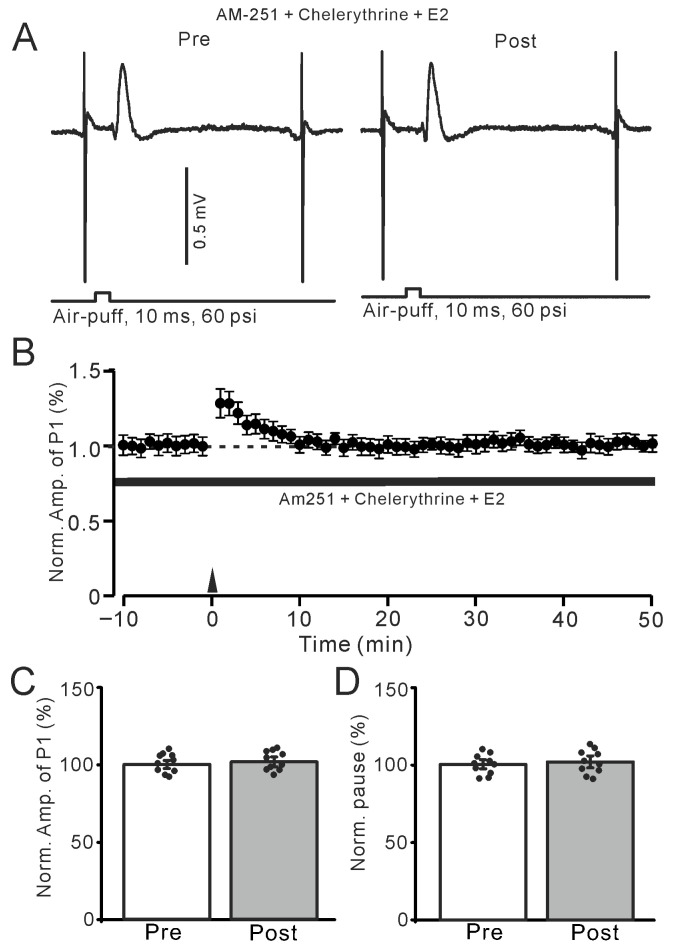
Inhibition of PKC abolished the E2-triggered facial stimulation-induced MLI-PC LTP. (**A**) Representative membrane potentials showing air-puff stimulation evoked MLI-PC response before (Pre) and after (Post) delivery of the 1 Hz (240 pulses) stimulation during treatment with a mixture of chelerythrine (5 μM) + AM-251 (5 μM) + E2 (100 nM). (**B**) A summary of data showing the normalized amplitude of P1 in the presence of chelerythrine + AM-251 + E2 before and after the air-puff stimuli train (black arrow). (**C**) The bar graph with individual data shows the normalized P1 amplitude before (Pre) and after (Post) 1 Hz stimulation during treatment with chelerythrine + AM-251 + E2 (Pre: 100.0 ± 2.5%, Post: 101.1 ± 3.1%, F (1,18) = 2.11, *p* = 0.22; *n* = 10 recordings/10 mice). (**D**) The bar graph with individual data shows the normalized SS pause time before (Pre) and after (Post) 1 Hz stimulation during treatment with chelerythrine + AM-251 + E2 (Pre: 100.0 ± 2.7%, Post: 101.6 ± 3.3%, F (1,18) = 3.12, *p* = 0.11; *n* = 10 recordings/10 mice). Note that inhibition of PKC abolished the E2-triggered MLI-PC LTD in the mouse cerebellar cortex in vivo.

We further used a specific membrane-permeable PKC inhibitor, chelerythrine chloride (5 μM), to investigate whether E2 triggers the MLI-PC LTP by activating the PKC signaling pathway. Inhibition of PKC with chelerythrine completely prevents the E2-triggered MLI-PC LTP (Figure 8A,B). In the presence of AM-251 and chelerythrine (5 μM), the amplitude of P1 was 101.1 ± 3.1% of that before the induction (*p* = 0.22; *n* = 10; Figure 8C), and the SS pause was 101.6 ± 3.3%of that before the induction (*p* = 0.11; *n* = 10; Figure 8D). The results indicate that with PKC inhibition, E2 could not trigger the 1 Hz facial stimulation-induced MLI-PC LTP, suggesting that the E2-triggered MLI-PC LTP is mediated by the PKC signaling pathway in vivo in mice.

**Figure 9:**
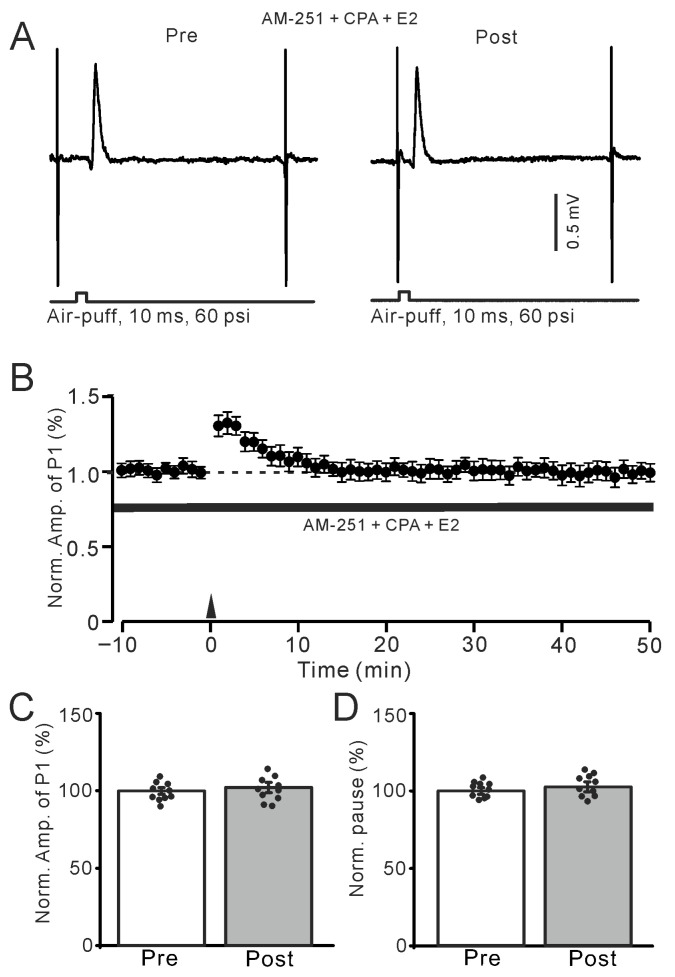
Depletion of intracellular Ca^2+^ abolished the E2-triggered MLI-PC LTP. (**A**) Representative membrane potentials show air-puff stimulation evoked MLI-PC synaptic response before (Pre) and after (Post) the delivery of 1 Hz stimulation during treatment with cyclopiazonic acid (CPA, 100 μM) + AM-251 (5 μM) + E2 (100 nM). (**B**) A summary of data showing the normalized amplitude of P1 in the presence of CPA + AM-251 + E2 before and after the air-puff stimuli train (black arrow). (**C**) The bar graph with individual data shows the normalized P1 amplitude before (Pre) and after (Post) 1 Hz stimulation during treatment with CPA + AM-251 + E2 (Pre: 100.0 ± 2.1%, Post: 100.8 ± 3.3%, F (1,18) = 2.06, *p* = 0.34; *n* = 10 recordings/10 mice). (**D**) The bar graph with individual data shows the normalized SS pause time before (Pre) and after (Post) 1 Hz stimulation during treatment with CPA + AM-251 + E2 (Pre: 100.0 ± 2.2%, Post: 101.3 ± 3.2%, F (1,18) = 3.22, *p* = 0.15; *n* = 10 recordings/10 mice). Note that depletion of intracellular Ca^2+^ completely prevented the E2-triggered MLI-PC LTD in the mouse cerebellar cortex in vivo.

**Figure 10:**
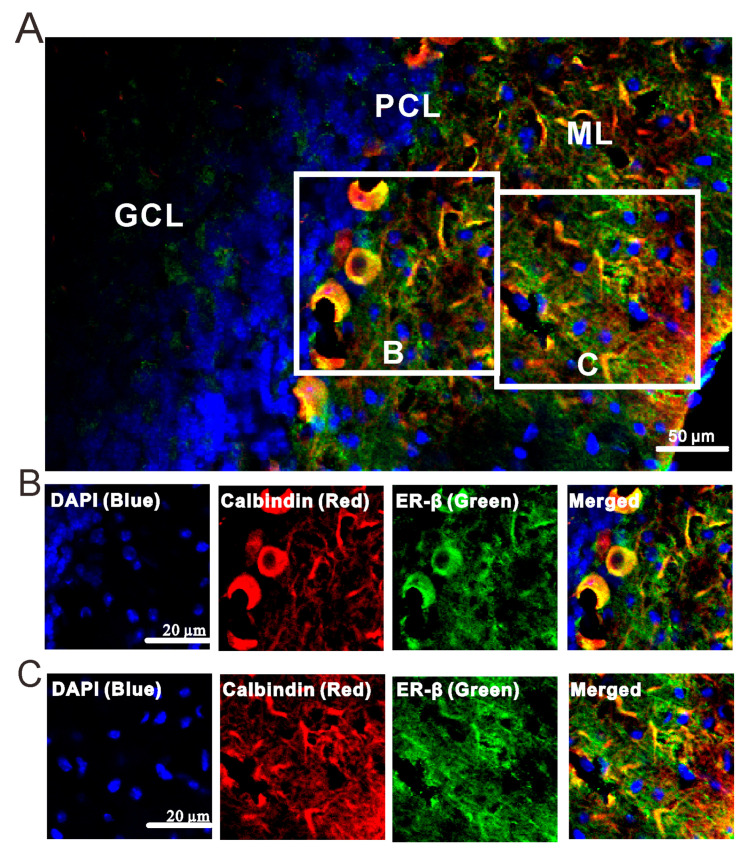
Expression of ERβ in the mouse cerebellar cortical Crus II. (**A**) The digital micrograph shows the confocal merged image of DAPI (blue), ERβ (green), and calbindin (red) in the mouse cerebellar Crus II. DAPI is a blue nucleic acid dye that preferentially dyes the dsDNA of cells. Calbindin is a marker for labeling PCs. (**B**) Higher magnifications of the boxed area (**B**) in (**A**), showing DAPI (blue), ERβ (green), calbindin (red), and merged immunofluorescence in the soma of PC. (**C**) Higher magnifications of the boxed area (**C**) in (**A**), showing DAPI (blue), ERβ (green), calbindin (red), and merged immunofluorescence in ML. The green ERβ immunoreactivity is expressed in dendrites and somas of PCs. ML, molecular layer; PCL, Purkinje cell layer; GCL, granular layer. Note that ERβ immunoreactivity was mostly expressed around the dendrites and somas of PCs in the cerebellar cortical Crus II.

We then observed whether the E2-triggered MLI-PC LTP requires the intracellular Ca^2+^ by cerebellar surface application of a calcium depletion, cyclopiazonic acid (CPA; 100 µM). For complete depletion of intracellular Ca^2+^, CPA (100 μM) was applied on the cerebellar surface for over 30 min. After depletion of intracellular Ca^2+^ and blockade of CB1R, E2 could not trigger the MLI-PC LTP induced by 1 Hz facial stimulation (Figure 9A,B). The amplitude of P1 was 100.8 ± 3.3% of that before the induction (*p* = 0.34; *n* = 10; Figure 9C). Meanwhile, the SS pause was 101.3 ± 3.2% of that before the induction (*p* = 0.15; *n* = 10; Figure 9D). The results indicate that with the depletion of intracellular Ca^2+^, E2 failed to trigger the facial stimulation-induced MLI-PC LTP in vivo in mice. Therefore, these results suggest that when E2 triggers the 1 Hz facial stimulation-induced MLI-PC LTP in the mouse cerebellar cortex, it requires intracellular Ca^2+^. In addition, consistent with a previous study [11], our immunohistochemistry results showed ERβ immunoreactivity in the cerebellar cortex (Figure 10A), which was mostly expressed around the dendrites and somas of PCs in the cerebellar cortical Crus II (Figure 10B,C). These results are consistent with the previous study [7], suggesting that E2 binds to ERβ, thereby triggering the facial stimulation-induced MLI-PC LTP in vivo in mice.

## 3. Discussion

We here demonstrated that facial stimulation induces MLI-PC LTD in mice, which is abolished by in vivo microinjection of E2 into the cerebellar molecular layer. This effect of E2 on facial stimulation-induced MLI-PC LTD is blocked by a selective AR antagonist. When CB1R-dependent MLI-PC LTD is inhibited, E2 triggers facial stimulation-induced MLI-PC LTP via ERβ activation. E2-triggered MLI-PC LTP is abolished by PKC inhibition or intracellular Ca^2+^ depletion (using cyclopiazonic acid), but not by PKA inhibition. These results indicate that E2 induces facial stimulation-induced MLI-PC LTP through the ERβ/PKC signaling cascade, which disrupts the induction of CB1R-dependent MLI-PC LTD in the mouse cerebellar cortex in vivo.

### 3.1. E2 Occludes the Facial Stimulation-Induced MLI-PC LTD via the Activation of ERs

Within the cerebellar cortex, both ERα and ERβ are widely expressed in the cerebellar cortex, especially in the granular cell layer and molecular layer. ERα is mainly localized in GCs [21,22], while high levels of ERβ are found in both granule cells and PCs [22,23,24,25,26]. Our previous data showed that ERβ immunoreactivity is expressed in the molecular layer and Purkinje cell (PC) layer, particularly around PC somas [7]. Notably, the present immunochemical data revealed that ERβ immunoreactivity was also predominantly expressed around the dendrites and somas of PCs in the cerebellar cortical Crus II. E2 has been shown to play a crucial role in regulating multiple physiological and cognitive processes, including reproductive functions, sexual behavior patterns, neural synaptic plasticity, and cognitive performance, as well as contributing to maintaining brain health and resilience via various ERs [6]. We recently demonstrated that E2 activates ERβ to modulate facial stimulation-evoked molecular layer interneuron (MLI)-PC synaptic transmission in the mouse cerebellar cortex [7]. E2 enhances NMDAR-mediated excitatory post-synaptic potentials and facilitates the hippocampal LTP in awake rats [13] and in rat hippocampal slices [14]. Estradiol targeting ERα or ERβ modulates synaptic transmission in the prefrontal cortex and hippocampus through various downstream signaling cascades in vitro in mice [27]. The activation of ERα triggers the emergence of LTP in GABAergic synaptic transmission within the oval bed nucleus of the stria-terminalis, and induces an NMDAR-dependent LTP at temporoammonic-hippocampal CA1 synapses in vitro in rats [15,16], whereas the activation of ERβ regulates the activity-dependent regulation of the dendritic spine cytoskeleton, hippocampal synaptic plasticity, and improves hippocampus-dependent cognition [11,12], and also potentiates parallel fiber–PC LTP in female mice [16]. Our data showed that the facial stimulation-induced MLI-PC LTD was completely prevented by microinjection of E2 into the cerebellar molecular layer in vivo in mice. The effect of E2 on the facial stimulation-induced MLI-PC LTD was completely prevented by the non-selective ERs antagonist, indicating that E2 occludes the facial stimulation-induced MLI-PC LTD in the mouse cerebellar cortex. These results suggest that E2 is secreted by cerebellar projecting estrogenergic neurons and released into the cerebellar molecular layer, contributing to the control of MLI-PC GABAergic plasticity induction and the modulation of motor behavior via the activation of ERs.

### 3.2. E2 Triggers the Facial Stimulation-Induced MLI-PC LTD by Activating ERβ

Previous studies using specific estrogen agonists have shown that the activation of ERα significantly enhances LTD in the CA1 region, while the activation of ERβ induces the inhibition of LTD in the CA1 region, implying that ERβ and ERα play opposite roles in the effect of E2 on synaptic plasticity in the CA1 region [11,12,15,16]. In the cerebellar cortex, E2 enhances the induction of the cerebellar PF-PC LTP, whereas it does not affect PF-PC LTD; it improves gain-decrease adaptation of the vestibulo-ocular reflex via activation of ERβ, as well as increases the density of PF-PC synapses, suggesting that estradiol can improve motor skills by potentiating cerebellar plasticity and PF-PC synapse formation in mice [16]. The present results showed that when blocking the CB1R-dependent MLI-PC LTD [5], E2 triggers 1 Hz facial stimulation-induced MLI-PC LTP in the mouse cerebellar cortex in vivo. Furthermore, the E2-triggered facial stimulation-induced MLI-PC LTP was not abolished by the selective ERα antagonist but by the selective ERβ blocker, suggesting that E2 may trigger the 1 Hz facial stimulation-induced MLI-PC LTP in the mouse cerebellar cortex by activating ERβ but not ERα. Moreover, pharmacological activation of ERβ triggers the MLI-PC LTP in the mouse cerebellar cortex, confirming that ERβ activation mediates the facial stimulation-evoked MLI-PC LTP in vivo in mice. These results suggest that E2 triggers the MLI-PC LTP via activating ERβ, resulting in an occlusion of MLI-PC LTD in the mouse cerebellar cortex.

### 3.3. E2 Triggers the Facial Stimulation-Induced MLI-PC LTD via the PKC/Ca^2+^ Signaling Cascade

Previous studies have demonstrated that E2 binds to cytoplasmic or membrane-associated ERs in the brain, thereby initiating intracellular signaling cascades involving several kinases, such as protein kinase A (PKA), protein kinase C (PKC), and Ca^2+^ influx [28]. In the present study, inhibition of PKA failed to prevent E2-triggered MLI-PC LTP in mouse cerebellar cortex in vivo, indicating that E2-triggered MLI-PC LTP is not mediated by the PKA signaling pathway. In addition, PKC activation has been shown to be required for LTP induction and persistence in the hippocampus [29,30,31,32], ventral tegmental area (VTA), and the cerebellar cortex [33]. In the hippocampus, earlier studies demonstrated that the inhibition of postsynaptic PKC prevents the induction and persistence of LTP in the hippocampal CA1 region, suggesting that PKC regulates LTP induction and maintenance starting immediately after LTP onset [29,30,34]. Additionally, inhibition of presynaptic PKC blocks the induction of NMDA receptor-dependent LTP, indicating that presynaptic PKC is essential for NMDA receptor-dependent LTP induction [31]. In the cerebellar cortex, it has been shown that PKC inhibition completely prevents sensory stimulation-induced, opioid-dependent MLI-PC LTD in vivo in mice [33]. Consistent with our previous findings [33], the present results demonstrate that PKC inhibition abolishes the E2-triggered MLI-PC LTP, suggesting that the E2-triggered MLI-PC LTP is dependent on the PKC signaling cascade. Moreover, E2 could directly induce an increase in intracellular calcium concentration by facilitating the influx of resting Ca^2+^ in the extracellular space, rather than by stimulating Ca^2+^ release from intracellular stores [35]. Our present data showed that with the depletion of intracellular calcium, E2 failed to trigger the facial stimulation-induced MLI-PC LTP, suggesting that for E2 to trigger the facial stimulation-induced MLI-PC LTP, it requires intracellular Ca^2+^ in the mouse cerebellar cortex in vivo. ERβ is expressed in the molecular layer and PC layer, indicating that ERβ expresses itself in dendrites and somas of both MLIs and PCs [11]. Therefore, E2 can bind to the ERβ of MLIs and PCs to trigger the facial stimulation-induced MLI-PC LTP via a presynaptic or postsynaptic PKC/Ca^2+^ signaling cascade.

### 3.4. Limitations and Further Directions

Since male mice were used in the study, the effect of E2 on facial stimulation-evoked MLI-PC synaptic plasticity remains unclear. Therefore, we intend to conduct a series of experiments in the future to investigate the mechanism underlying the effect of E2 on long-term synaptic plasticity of MLI-PC in the cerebellar cortex of female mice across different estrous cycle stages, and analyze whether peripheral estrogen levels are involved in the regulation of central E2 on MLI-PC long-term synaptic plasticity. Second, the effect of E2 on facial stimulation-induced MLI-PC LTP was examined in urethane-anesthetized mice, and we cannot completely rule out the possible effects of the anesthetic on facial stimulation-induced MLI-PC synaptic plasticity. Third, the present results were mainly obtained from in vivo electrophysiological recordings, which showed that E2 occludes the expression of MLI-PC LTD by triggering MLI-PC LTP in the mouse cerebellar cortex. However, whether the effect of E2 on facial stimulation-induced MLI-PC synaptic plasticity is correlated with locomotor behavior remains unknown. We will attempt to investigate the effects of E2 on the firing activity of MLI and the synaptic plasticity of MLI-PC in awake, freely moving mice, as well as explore the mechanism underlying the influence of E2 on the locomotor behavior of the free movement mice.

## 4. Materials and Methods

### 4.1. Animal Anesthesia and Surgical Procedures

The anesthesia and surgical procedures have been described previously [36,37]. In brief, the experimental procedures were approved by the Animal Care and Use Committee of Yanbian University and are in accordance with the animal welfare guidelines of the U.S. National Institutes of Health. The permit number is SYXK (Ji) 2025-006, Approval Date: 11 July 2025. In order to avoid potential interference from hormonal fluctuations associated with the estrous cycle of female mice, only male adult C57BL6/J mice (6–8 weeks old) were employed in this study. The mice were housed under a 12 h light and a 12 h dark cycle with free access to food and water in a colony room under constant temperature (23 ± 1 °C) and humidity (50 ± 5%). Mice (*n* = 120) were anesthetized with urethane (1.3 g/kg body weight i.p.) and were tracheotomized to avoid respiratory obstruction. A watertight chamber was created, and a 1–1.5 mm craniotomy was drilled to expose the cerebellar surface corresponding to Crus II. The cerebellar surface was constantly superfused with oxygenated artificial cerebrospinal fluid (ACSF: 125 mM NaCl, 3 mM KCl, 1 mM MgSO_4_, 2 mM CaCl_2_, 1 mM NaH_2_PO_4_, 25 mM NaHCO_3_, and 10 mM D-glucose) with a peristaltic pump (Gilson Minipulse 3; Villiers, Le Bel, France) at 0.5 mL/min. Rectal temperature was monitored and maintained at 37.0 ± 0.2 °C using a body temperature controller.

### 4.2. Electrophysiological Recording and Induction of MLI-PC LTD

Cell-attached recordings were performed on the PCs in the cerebellum using an Axopatch-700B patch-clamp amplifier (Molecular Devices, LLC, San Jose, CA, USA). The recording electrodes were fabricated using a PB-10 automatic pipette puller (Narishige, Tokyo, Japan), with thin glass tubes (1.5 mm diameter) serving as the electrode material. Internally, the recording electrodes were filled with 10–20 μL of artificial cerebrospinal fluid (ASCF), and their resistance was adjusted to a range of 3–5 mΩ. A micromanipulator (Sutter, Novato, CA, USA) was employed to control the recording electrodes, and their position was precisely adjusted under a Nikon Eclipse FN1 microscope (Nikon, Tokyo, Japan). The recording electrode was carefully inserted to a depth of 150–200 μm beneath the pia mater until it reached the Purkinje cell layer (PCL). The spontaneous firing activity signals of the PCs were collected via a Digidata 1550B analog-to-digital converter (Molecular Devices, LLC, San Jose, CA, USA) and the Clampex11.2 software. PCs were identified by observing the characteristic firing pattern, which consisted of regular simple spikes (SSs) interspersed with irregular complex spikes (CSs). In addition, another glass electrode filled with E2 (100 nM) was placed on the surface of the cerebellar molecular layer, 100–150 μm away from the recording electrode for PCs. An air puff applied to the ipsilateral whisker pad evoked a response in cerebellar Purkinje cells (PCs), which manifested as a small negative component (N1), followed by a large positive component (P1), and a pause in simple spike (SS) firing (Figure 1A). Based on our previous studies, N1 corresponds to the parallel fiber volley, while P1 is identified as the GABAergic inhibitory synaptic transmission between molecular layer interneurons (MLIs) and PCs [5,7,19,33]. The stimulation protocol for inducing the long-term plasticity of MLI-PC synapses was demonstrated previously [5,19,33] (Supplementary Materials).

### 4.3. Immunohistochemistry and Imaging

Immunohistochemistry and imaging have been demonstrated in a previous study [7]. Adult male C57BL6/J mice (*n* = 3) were deeply anesthetized with an intraperitoneal injection of 2,2,2-tribromoethanol (250 mg/kg) and then transcardially perfused with cold phosphate buffer (PBS) followed by PFA PBS solution. The brain was postfixed in 4% PFA for 48 h at 4 °C and was exposed to 10% sucrose, 20% sucrose, and 30% sucrose in PBS. Then, the cerebellum was sectioned into 8 μM slices in the sagittal plane using a freezing microtome (CM1900, Leica Microsystems GmbH, Wetzlar, Germany). The sections were attached to microscope slides. The slides were permeabilized with 0.3% Triton X-100 in PBS and were blocked (10% donkey serum in PBS), and further incubated in a primary antibody (rabbit anti-ERβ, 1:1000; Sigma-Aldrich, Shanghai, China; mouse anti-calbindin-D28k, 1:400; proteintech), followed by donkey anti-rabbit DyLight™ 488 (Invitrogen, Carlsbad, CA, USA, 1:500), goat anti-mouse Cyanine 5 (Invitrogen, Carlsbad, CA, USA, 1:500) and 4′,6-diamidino-2-phenylindole (DAPI, 1:1000). The incubation time for the primary antibody was overnight at 4 °C. For the secondary antibody and DAPI, the incubation time was 2 h at room temperature. The sections were then washed three times in PBS and dried slightly, followed by sealing with anti-fade mounting medium. Fluorescence images were acquired using a high-speed multiphoton confocal laser-scanning microscope (A1RMP, Nikon, Tokyo, Japan).

### 4.4. Chemicals

The reagents included β-estradiol, ICI182780 (a selective ERα/ERβ antagonist), MPP dihydrochloride (a selective ERα antagonist), PHTPP (a selective ERβ antagonist), DPN (a highly potent β-ER agonist), and Cyclopiazonic acid (CPA, a specific inhibitor of the sarco-endoplasmic reticulum Ca^2+^-ATPases), and were purchased from GLPBIO (Montclair, CA, USA). KT5720 and Chelerythrine Chloride were bought from Tocris (Cookson Electronics Limited, London, UK). E2 was dissolved in ACSF and micro-applied onto the molecular layer above the recorded PCs at 0.1 μL/sec for 100 s by a micro pump (KDS-210, KD Scientific, Holliston, MA, USA). The other drugs were finally dissolved in ACSF, and the bath was applied directly onto the cerebellar surface by a peristaltic pump (Gilson Minipulse 3; Villiers, Le Bel, France) at 0.5 mL/min. For complete inhibition of PKA and PKC, KT5720 and Chelerythrine Chloride were applied onto the cerebellar surface 20 min before the electrical recordings started. After a stable cell attachment was configured, the baseline was recorded for 100 s, then the perfusion of chemicals was initiated.

### 4.5. Statistical Analysis

Electrophysiological data were analyzed with Clampfit 11.2 (Molecular Devices, Foster City, CA, USA). The amplitude of P1 was measured from baseline to the peak of P1, and the pause of SS was the time measured from the peak of P1 to the first SS after the air-puff stimulation (Figure 1A). All the electrophysiological data were normalized to baseline and used for further analyses. All the data were maintained constant for an individual recorded neuron in treatments of ACSF, drugs, and recovery. Post-stimulation values were calculated from 40–50 min after the induction stimulation. For each recording, the normalized amplitudes of P1 before (Pre) and after the induction stimulation (Post) were divided by the mean value of all baseline measurements (pre-stimulation) in each group, then multiplied by 100. In all experiments, the normality of the data was examined using the Kolmogorov–Smirnov test, and parametric or non-parametric statistics were employed accordingly. For within-group comparisons of Post vs. Pre data, statistical significance was determined using one-way ANOVA with post hoc multiple comparisons (Figure 1, Figure 2 and Figure 4C,D, Figure 5C,D, Figure 6C,D, Figure 7C,D, Figure 8C,D and Figure 9C,D, respectively). For between-group comparisons of Post data, two-way ANOVA followed by Tukey’s post hoc tests (Figure 3C,D) (SPSS, Chicago, IL, USA) was employed. Values are expressed as the mean ± S.E.M. *p*-values below 0.05 were considered to indicate a statistically significant difference between experimental groups.

## 5. Conclusions

The present study demonstrates that 1 Hz facial stimulation induces MLI-PC LTD in the mouse cerebellar cortex via activation of the CB1 receptor, suggesting that MLI-PC LTD plays a critical role in motor learning. Importantly, E2 reverses CB1 receptor-mediated MLI-PC LTD by activating ERβ via the PKC/Ca^2+^ signaling cascade, indicating that E2 modulates motor learning behavior by switching CB1 receptor-mediated MLI-PC LTD in vivo in mice. These results provide novel evidence for understanding the mechanism by which E2 modulates somatosensory stimulation-induced cerebellar MLI-PC inhibitory synaptic plasticity and its implications for motor learning in rodents.

## Data Availability

The datasets generated and analyzed during the current study are available from the corresponding author on reasonable request.

## Supplementary Materials

The following supporting information can be downloaded at: https://www.mdpi.com/article/10.3390/ijms26209973/s1.
