# Supplementary material for: Estradiol Triggers Cerebellar MLI-PC LTP via ERβ/Protein Kinase C Signaling Cascades in Mice In Vivo
Source: Int J Mol Sci. 2025 Oct 14;26(20):9973. doi: 10.3390/ijms26209973 (PMC12562853; doi:10.3390/ijms26209973)
Supplement: Supplementary file 1 [file ijms-26-09973-s001.zip › ijms-3883325-supplementary.pdf]

Stimulation protocol

The test stimulus was a 10 ms air-puff stimulation at 60 psi. The inducing stimulus was a 1 Hz stimulation with 240 pulses, designed to induce the long-term depression (LTD) of the MLI - PC synapses. Initially, recordings were made in the test mode for 10 minutes. Once the baseline had stabilized, the inducing air-puff stimulation was applied, and the recording was maintained for at least 50 minutes.
